# Proteome profiling of evolved methicillin-resistant *Staphylococcus aureus* strains with distinct daptomycin tolerance and resistance phenotypes

**DOI:** 10.3389/fmicb.2022.970146

**Published:** 2022-08-04

**Authors:** Jordy Evan Sulaiman, Lexin Long, Pei-Yuan Qian, Henry Lam

**Affiliations:** ^1^Department of Chemical and Biological Engineering, The Hong Kong University of Science and Technology, Kowloon, Hong Kong SAR, China; ^2^Department of Ocean Science and Hong Kong Branch of Southern Marine Science and Engineering Guangdong Laboratory (Guangzhou), The Hong Kong University of Science and Technology, Kowloon, Hong Kong SAR, China; ^3^Southern Marine Science and Engineering Guangdong Laboratory, Guangzhou, China

**Keywords:** MRSA, antibiotics, daptomycin, evolution, tolerance, resistance, proteomics

## Abstract

Methicillin-resistant *Staphylococcus aureus* (MRSA) is a highly dangerous pathogen, and daptomycin has been increasingly used to treat its infections in clinics. Recently, several groups have shown that tolerance and resistance of microbes can evolve rapidly under cyclic antibiotic exposure. We have previously shown that the same tolerance and resistance development occurs in MRSA treated with daptomycin in an adaptive laboratory evolution (ALE) experiment. In the present study, we performed proteomic analysis to compare six daptomycin-tolerant and resistant MRSA strains that were evolved from the same ancestral strain. The strain with a higher tolerance level than the others had the most different proteome and response to antibiotic treatment, resembling those observed in persister cells, which are small subpopulations of bacteria that survive lethal antibiotics treatment. By comparing the proteome changes across strains with similar phenotypes, we identified the key proteins that play important roles in daptomycin tolerance and resistance in MRSA. We selected two candidates to be confirmed by gene overexpression analysis. Overexpression of EcsA1 and FabG, which were up-regulated in all of the tolerant evolved strains, led to increased daptomycin tolerance in wild-type MRSA. The proteomics data also suggested that cell wall modulations were implicated in both resistance and tolerance, but in different ways. While the resistant strains had peptidoglycan changes and a more positive surface charge to directly repel daptomycin, the tolerant strains possessed different cell wall changes that do not involve the peptidoglycan nor alterations of the surface charge. Overall, our study showed the differential proteome profiles among multiple tolerant and resistant strains, pinpointed the key proteins for the two phenotypes and revealed the differences in cell wall modulations between the daptomycin-tolerant/resistant strains.

## Introduction

In the past 20 years, *Staphylococcus aureus* infections have become more dangerous and expensive to treat owing to the increased prevalence of antimicrobial resistance (Neyra et al., 2014). Methicillin-resistant *S. aureus* (MRSA) is one of the most dangerous pathogens to date, causing infections in both high-risk patients in hospitals (hospital-associated MRSA) and in healthy, non-hospitalized individuals without risk factors (community-associated MRSA). Since 2001, the increase in MRSA exposures and infections in the United States was largely attributed to the community-associated strains because they cannot be controlled solely based on measures implemented within the health care settings (Como-Sabetti et al., 2009; Stefani et al., 2012). Several studies have reported that MRSA was the most common cause of skin and soft tissue infections in hospitals (King et al., 2006; Moran et al., 2006). In Europe, approximately 20% of *S. aureus* isolates were methicillin-resistant, whereas in the United States, the prevalence of MRSA was more than 50% (System, 2004). MRSA infections are harder to treat than ordinary *S. aureus i*nfections because they are resistant to many types of *antibiotics*. Two of the most frequently used last-resort antibiotics to treat MRSA infections are vancomycin and daptomycin, with the former being the first choice. However, due to the excessive use of vancomycin, there have been multiple reports of MRSA isolates with increased vancomycin minimum inhibitory concentration (MIC), hence making daptomycin a more attractive treatment option (Murray et al., 2013).

Bacterial populations can adapt to stresses and a wide range of treatment conditions, including antibiotic therapy. Through *in vitro* laboratory evolution, several groups have shown that tolerance and resistance evolved rapidly under frequent, cyclic antibiotic treatment (Fridman et al., 2014; Mechler et al., 2015; Van den Bergh et al., 2016; Khare and Tavazoie, 2020; Sulaiman and Lam, 2020a,b, 2021b; Sulaiman et al., 2021). More recently, Liu et al. showed that this development of tolerance and resistance also occurs in patients with MRSA infection receiving drug combinations of daptomycin and rifampin (Liu et al., 2020). Resistance and tolerance are two different bacterial adaptation strategies against antibiotics. While resistance allows bacteria to grow at an elevated antibiotic concentration, tolerance describes the ability of a population to survive, but not grow, under lethal antibiotic concentrations for an extended period. Recently, it was suggested that tolerance facilitates the development of resistance (Levin-Reisman et al., 2017, 2019; Santi et al., 2021; Sulaiman and Lam, 2021a). Therefore, combatting tolerance is key to stopping the development of resistance (Windels et al., 2019), and a more in-depth investigation of the key players and pathways responsible for various tolerance phenotypes (the “tolerome”) is necessary (Brauner et al., 2016; Sulaiman and Lam, 2021a). Unlike resistance that directly counteracts the action mechanism of the antibiotic, tolerance is thought to arise from a perturbed biological network of multiple pathways. Thus, proteomics is the most suitable tool to inspect the mechanisms of tolerance and to highlight the key players responsible for the phenotype (Sulaiman and Lam, 2019; Zhang et al., 2020). Proteomics has been proven to be useful in revealing the key players in *E. coli* persistence and tolerance to various antibiotics (Hu et al., 2015; Sulaiman et al., 2018; Sulaiman and Lam, 2020a,b), and has also been used to investigate persistence and tolerance phenotypes in *S. aureus* (Chatterjee et al., 2009; Overton et al., 2011; Conlon et al., 2013; Conlon et al., 2016; Zalis et al., 2019; Huemer et al., 2021; Sulaiman et al., 2021, 2022). Persistence is a phenotype similar to tolerance, but unlike tolerance where most of the cells within the population are tolerant to the drug, persistence describes a situation where the tolerant cells only occur in a small subpopulation, called “persisters” (Sulaiman and Lam, 2021a, 2022).

Recently, our group performed adaptive laboratory evolution (ALE) experiments on MRSA using daptomycin and generated strains with distinct tolerance and resistance phenotypes (Sulaiman and Lam, 2021b). All of the daptomycin-resistant mutants have a single point mutation in the *mprF* gene but in different locations. Various mutations in the *mprF* gene, as well as in the *walKR* and *dlt* operon genes, have been frequently observed in clinical isolates of MRSA and extensively studied (Tran et al., 2015). Mainly, small nucleotide polymorphisms (SNPs) in the *mprF* gene were thought to increase either the LysPG synthase activity, the flippase activity, or both, leading to an increased level of LysPG in the outer membrane leaflet, which could increase the electrostatic repulsion of daptomycin and other cationic antimicrobial peptides (CAMPs; Ernst and Peschel, 2011; Bayer et al., 2013). Ernst et al. have recently reviewed the current knowledge of MprF-mediated daptomycin resistance in *S. aureus* (Ernst and Peschel, 2019). In contrast, the newly discovered tolerant mutants bear single point mutations in genes unrelated to resistance and have not been previously reported to cause decreased susceptibility to antibiotics. Interestingly, these mutations led to different levels of tolerance toward daptomycin, with one strain (TOL6) exhibiting a much higher survival than the other strains (over 100-fold increase in survival after 3 h of daptomycin treatment). In the present study, we compared the proteomes of multiple daptomycin-tolerant and resistant MRSA strains that were evolved from the same ancestral strain and thus bearing minimal changes in the genotype. Using this strategy, we searched for any commonalities in terms of up-regulated and down-regulated processes or pathways between multiple resistant and tolerant strains. Then, we verified the importance of two DEPs common in the tolerant strains through gene overexpression analysis. Moreover, through various assays that assess cell wall properties, we revealed that the tolerant and resistant strains had distinct modifications in their cell wall. Although it was known that daptomycin did not directly inhibit cell wall synthesis, our study showed that changes in the cell wall properties were commonly observed in daptomycin-tolerant and resistant strains, and provided evidence that such changes may affect *S. aureus* susceptibility toward daptomycin (Gray and Wenzel, 2020).

## Materials and methods

### Bacterial strains and growth conditions

Bacterial strains used in this study are methicillin-resistant *S. aureus* (MRSA) ATCC 43300 and daptomycin-tolerant (TOL2, TOL5, TOL6) and resistant (RES1, RES2, RES3) MRSA strains. For our experiments, exponential phase cultures were prepared by incubating a 1:200 diluted overnight culture in cation-adjusted Mueller-Hinton (MH) broth until OD_600_ reached ~0.1 at 37°C with shaking. MH broth used in this study is supplemented with Ca^2+^ to a final concentration of 50 mg/l to mimic the physiological levels of calcium ions, which is important for the concentration-dependent bactericidal activity of daptomycin (Silverman et al., 2003; Safdar et al., 2004; Steenbergen et al., 2005). MH agar was used for colony counts.

The tolerant and resistant MRSA strains were obtained from a recent adaptive laboratory evolution (ALE) experiment using daptomycin antibiotic (Sulaiman and Lam, 2021b). Briefly, to generate the evolved strains, MRSA ATCC 43300 is used as the ancestral strain for the evolution experiment. Either exponential or stationary phase culture was exposed to 10 mg/l daptomycin (1 h for the exponential phase culture and 3 h for the stationary phase culture), and the antibiotic-containing medium was removed by washing three times in MH broth. Finally, the cells were resuspended in 1 ml fresh MH and grown overnight at 37°C. The next day, 3 μl of the overnight culture was resuspended in 1 ml fresh MH and grown to either exponential or stationary phase, and the antibiotic treatment was repeated. The evolved strains (TOL2, TOL5, TOL6, RES1, RES2, and RES3) were isolates collected from different lineages at different time points during the evolution experiments. The list of single point mutations in the evolved strains is summarized in Supplementary Table S1. Whole-genome sequence data of the evolved strains is available in the BioProject database (NCBI) under the accession number PRJNA724993.

### Tolerance and resistance assay

To measure the tolerance level of the different strains, we measured the time-kill curve under daptomycin treatment (10 mg/l). For susceptibility assay toward vancomycin, the concentration used for treatment is 30 mg/l. To assess cell viability after antibiotic treatment, the number of survivors were counted by serially diluting cultures in MH broth, plating 100 μl on MH agar and spread plates.

The MICs of the population were recorded by the broth macrodilution method (Wiegand et al., 2008). The MIC was determined by incubating ~$5.10^{5}$ exponential phase culture in MH medium for 16 h with various concentrations of antibiotics (daptomycin or vancomycin), and inhibition of growth was observed based on the lack of turbidity. The MIC value was determined as the lowest concentration without growth, according to EUCAST guidelines. Experiments were performed with three independent cultures.

### Lysostaphin lysis assay

Lysostaphin lysis assay was performed following protocols described in literature with a slight modification (Gründling et al., 2006; Barros et al., 2019). Cells were grown to an OD_600_ ~ 0.6 and harvested by centrifugation. Cells were washed with water and resuspended in PBS supplemented with 5 mg/l lysostaphin (Sigma Aldrich). Cells were then incubated at 37°C and the decrease in OD_600_ was monitored over time.

### Cytochrome C binding assay

The relative positive surface charge of *S. aureus* strains was determined by quantifying the association of the positively charged cytochrome *c* (Sigma) to the staphylococcal surface (Berti et al., 2015). The cytochrome *c* binding assay was performed following protocols from previous literature (Mehta et al., 2012; Gasch et al., 2013). Briefly, 1:1000 of overnight cultures was grown in fresh medium to logarithmic phase. Cells were harvested, washed twice with MOPS (morpholinepropanesulfonic acid) buffer (20 mM, pH 7.0), and the bacterial suspension was adjusted to an OD_600_ of ~1. Aliquots of 1 ml were centrifuged, and the cell pellets were resuspended in 200 μl MOPS buffer and 50 μl of cytochrome *c* solution was added (equine heart, 2.5 mg/ml in MOPS buffer; Sigma). Samples were incubated for 10 min at room temperature, separated by centrifugation, and the supernatants were recovered. The amount of cytochrome *c* remaining in the supernatant after a 10-min binding interaction with *S. aureus* cells was quantified spectrophotometrically at an optical density at 530 nm (OD_530_). The more unbound cytochrome *c* is detected in the supernatant suggest that the surface charge is more positive.

### Sample preparation for proteomics

For proteomics analysis, exponential phase ancestral strain, tolerant (TOL2, TOL5, TOL6) and resistant strains (RES1, RES2, RES3) were treated with sub-MIC doses of daptomycin (0.25 mg/l) for 1 h, which should enable the populations to elicit an antibiotic response (Liu et al., 2014, 2016; Sulaiman et al., 2021). Exponential phase cells before antibiotic treatment were also collected. Similar to our previous work (Sulaiman et al., 2021), two different strategies are used for the proteomics analysis: (i) First, the proteome profile of the evolved strains was compared to the ancestral strain as a control to reveal the effect of the point mutations on the phenotype of the tolerant/resistant strains. (ii) Next, we compared the proteome profile of each strain before and after antibiotic treatment to obtain strain-specific antibiotic response toward sub-inhibitory daptomycin exposure. For all proteomics experiments, three biological replicates were performed for each sample including the control sample.

The cell pellet was suspended in 300 μl of lysis buffer (8 M Urea, 50 mM Tris–HCl pH 8.0), frozen in liquid nitrogen, and sonicated for 10 min. The sample was centrifuged (16,000 × *g* for 10 min) to remove cell debris and insoluble materials. An aliquot of the sample was taken for BCA protein assay (Pierce™ BCA Protein Assay Kit). After protein quantification, the sample was reduced by dithiothreitol (DTT; 0.1 M final concentration) at 37°C for 1 h. For shotgun proteomics, 150 μg of proteins were mixed with up to 250 μl of the exchange buffer (6 M Urea, 50 mM Tris–HCl pH 8.0, 600 mM guanidine HCl), transferred to Amicon® filter device (Millipore, Darmstadt, Germany), and centrifuged (14,000 × *g* for 20 min). The proteins in the filter device were alkylated with iodoacetamide (IAA, 50 mM in exchange buffer) in dark for 20 min, and then centrifuged (14,000 × *g* for 20 min). To reduce the urea concentration, 250 μl of 50 mM ammonium bicarbonate was added to the filter device and centrifuged (14,000 × *g* for 20 min). This step was repeated once. Proteins were digested by sequencing-grade modified trypsin (1:50 w/w, Promega, Madison, WI) for 12 h at 37°C. Then, the sample was acidified with 10% formic acid to a final concentration of 0.1% (v/v) and centrifuged for 16,000 × *g* for 5 min. Finally, the samples were desalted by C18 reverse-phase ZipTip (Millipore, Darmstadt, Germany) and dried with SpeedVac (Eppendorf, Hamburg, Germany) for 30 min.

### Liquid chromatography

The samples were reconstituted in 25 μl water/acetonitrile/formic acid in a 97.9:2:0.1 ratio (v/v/v), and processed through Bruker nanoElute Ultra-High-Performance Liquid Chromatography (UHPLC; Bruker Daltonics, Bremen, Germany) coupled to a hybrid trapped ion mobility-quadrupole time-of-flight mass spectrometer (TimsTOF Pro, Bruker Daltonics, Bremen, Germany) *via* a nano-electrospray ion source (Captive Spray, Bruker Daltonics). A volume of 1 μl (approximately 200 ng of the protein digest) was injected into the UHPLC system and separated on an IonOpticks 25 cm Aurora Series Emitter column with Captive Spray Insert (250 mm × 75 μm internal diameter, 120 Å pore size, 1.6 μm particle size C18) at a flow rate of 0.3 μl/min. The mobile phase composition is 0.1% formic acid in water for solvent A, and 0.1% formic acid in acetonitrile for solvent B. The gradient was applied from 2 to 5% of solvent B for 0.5 min, from 5 to 30% of solvent B for 26.5 min, and then from 30 to 95% of solvent B for 0.5 min. In the end, the mobile phase was kept at 95% of solvent B for 0.5 min, and then decreased to 2% of solvent B for 0.1 min. 2 min equilibration with 2% of solvent B was applied before the next injection.

### Mass spectrometry

A detailed description of the Bruker TimsTOF Pro mass spectrometer used in this work can be found in the literature (Meier et al., 2015, 2018). We set the accumulation and ramp time to 100 ms each and recorded mass spectra in the range from m/z 100–1700 using the positive electrospray mode. The ion mobility was scanned from 0.85 to 1.30 *Vs*/cm^2^. The quadrupole isolation width was set to 2 Th for m/z < 700 and 3 Th for m/z > 700, and the collision energy was linearly increased from 27 eV to 45 eV as a function of increasing ion mobility. The overall acquisition cycle of 0.53 s comprised one full TIMS-MS scan and four Parallel Accumulation-Serial Fragmentation (PASEF) MS/ MS scans. Low-abundance precursor ions with an intensity above a threshold of 2,500 counts but below a target value of 20,000 counts were repeatedly scheduled and otherwise dynamically excluded for 0.4 min. The TIMS dimension was calibrated linearly using three selected ions from the Agilent ESI LC/MS tuning mix [m/z, 1/K0: (622.0289, 0.9848 *Vs* cm^−2^), (922.0097, 1.1895 *Vs* cm^−2^), (1221,9906, 1.3820 *Vs* cm^−2^)] in positive mode.

### Sequence database searching of proteomics data

The raw data were converted to mgf files by Bruker Compass DataAnalysis (version 5.2), and subsequently converted to mzML files by msconvert of the ProteoWizard (version 3.0.20229 64-bit; Kessner et al., 2008). The mzML files were searched using Comet (version 2016.01 rev.2; Eng et al., 2013) with a custom database. Briefly, the genome sequence of *S. aureus* ATCC 43300 was converted into a protein database using the gene prediction tool GeneMark (version 3.25; Lukashin and Borodovsky, 1998). The proteins were then annotated using BLASTp (version 2.7.1) from NCBI using *S. aureus* NCTC 8325 as the protein database. The sequences of common contaminants, such as trypsin and human keratins, and decoy sequences generated by shuffling amino acid sequences between tryptic cleavage sites were added to the database. The decoy sequences in the database are used for the false discovery rate (FDR) estimation of the identified peptides. The search parameters criteria were set as follows: 40 ppm peptide mass tolerance, monoisotopic mass type, fully digested enzyme termini, 0.05 amu fragment bin tolerance, 0 amu fragment bin offset, carbamidomethylated cysteine, and oxidated methionine as the fixed and variable modifications, respectively. The search results from Comet were processed by PeptideProphet (Keller et al., 2002), iProphet, and ProteinProphet of the Trans-Proteomics Pipeline (TPP; Deutsch et al., 2010) in the decoy-assisted non-parametric mode. Every mzML run was analyzed independently. Protein identifications were filtered at a false discovery rate of 0.01 as predicted by ProteinProphet. The mass spectrometry proteomics data have been deposited to ProteomeXchange via the PRIDE repository with the dataset identifier PXD026741.

### Label-free quantification of proteomics data by spectral counting

The proteins identified in at least two out of three biological replicates were used for label-free quantification by spectral counting. The quantification of proteins was given by the normalized spectral abundance factor (NSAF; Paoletti et al., 2006), where the number of peptide-spectrum matches (PSMs) for each protein divided by the length of the corresponding protein is normalized to the total number of PSMs divided by the lengths of protein for all identified proteins. The differentially expressed proteins were filtered by the following cutoff: average spectral counts of at least three, the *p* value for Student’s *t-*test on the NSAF values were lower than 0.05, and the fold changes were higher or lower than ±1.5-folds.

### Bioinformatics analysis

We visualize our proteomic data using principal component analysis (PCA) of the log NSAF values using the PCA function from the sklearn package with centering and scaling in python. We added 95% confidence intervals by calculating correlation matrices for the three replicates of each sample and then adding these intervals to our plot using the matplotlib package in python. To compare the protein expression profiles between different populations, we generated a heat map of fold changes of the differentially expressed proteins identified across the ancestral strain and evolved strains using the in-house scripts. To highlight potentially important proteins among the differentially expressed proteins, STRING version 11.0 (Szklarczyk et al., 2016) was used to predict the protein–protein interactions and to visualize the interactions. DAVID (Database for Annotation, Visualization and Integrated Discovery) version 6.8 (Sherman and Lempicki, 2009) was used for gene ontology (GO) and pathway analysis.

### Gene overexpression of the differentially expressed proteins

Gene overexpression was accomplished using a tetracycline-inducible expression vector pRMC2. The bacterial strains, plasmids, and primers used in this study are listed in Supplementary Table S6. The plasmid pRMC2 was obtained from Tim Foster (Corrigan and Foster, 2009; Addgene plasmid #68940^1^ 68,940).

Briefly, competent cells were first prepared as previously described and stored at −80°C (Chen et al., 2017). Then, the constructed plasmid was electroporated into the wild-type MRSA ATCC 43300 strain by thawing 50 μl of competent cells on ice for 10 min, mixing it with 1–2 μg of the plasmid, and transferring them into a 1 mm electroporation cuvette (Bio-Rad, Hercules, CA, USA). Cells were pulsed at 2.5 kV, 100 Ω, and 25 μF, incubated in 1 ml of tryptic soy broth (TSB) at 30°C for 1 h, and followed by plating on a TSB agar plate containing 7.5 μg/ml chloramphenicol for screening. Mutant strains were then subjected to relevant tolerance and resistance assays.

### Real-time quantitative PCR

Real-time Quantitative PCR (RT-qPCR) was performed to confirm the overexpression of the selected genes. The primers for real-time PCR are listed in Supplementary Table S6. Briefly, a 3 ml overnight culture of mutant MRSA strains (with the addition of 0.2 μg/ml anhydrotetracycline) was harvested, stabilized with RNAprotect Bacteria Reagent (Qiagen, Hilden, German), and total RNA was extracted with RNeasy PowerBiofilm Kit (Qiagen, Hilden, German) according to the manufacturer’s instruction. The RNA was first reverse transcribed to cDNA with RevertAid H Minus First-Strand cDNA Synthesis Kit after the removal of genomic DNA using DNase I (Thermo Fisher Scientific Inc., Waltham, MA, USA), followed by quantification on a Roche Diagnostics GmbH LightCycler 480 Instrument II Realtime PCR System using SYBR Green RT-PCR Reagents Kit (Applied Biosystems) with the following procedures: (i) polymerase activation at 95°C for 10 min, and (ii) annealing and extension at 53°C for 1 min with a total of 40 cycles. The specificity of primer pairs for the PCR amplification was checked by the melting curve analysis which was performed immediately after amplification. Two biological replicates and two technical replicates were performed for each sample, and the relative gene expression level was calculated based on the 2^−ΔΔCt^ method using *gyrA* as the internal-reference gene (Livak and Schmittgen, 2001). The RT-qPCR validation results is shown in Supplementary Figure S3.

## Results and discussion

### Proteome profiling of MRSA with distinct daptomycin tolerance and resistance phenotypes

**Figure 1:**
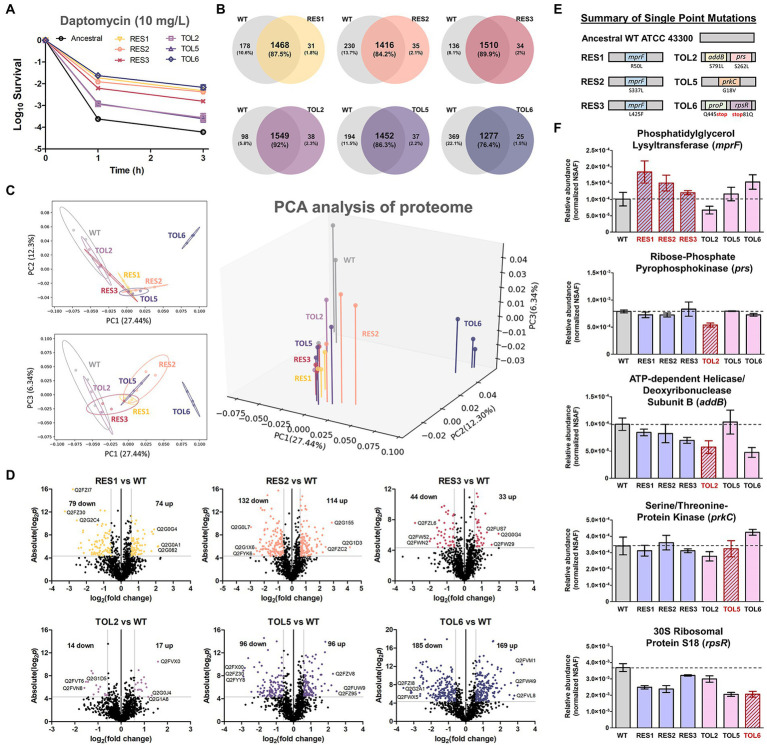
Proteome profile comparison between the tolerant/resistant strains and the ancestral strain. **(A)** Time-kill curve of the ancestral strain, resistant strains (RES1, RES2, RES3), and tolerant strains (TOL2, TOL5, TOL6) upon daptomycin treatment (10 mg/l) for 3 h (mean ± s.e.m., *n* = 3). **(B)** Venn diagrams for proteome comparison of the resistant strains (RES1, RES2, RES3) and tolerant strains (TOL2, TOL5, TOL6) with the ancestral strain. **(C)** Principal Component Analysis (PCA) of proteomes of the tolerant/resistant strains and the ancestral strain. Projections of PC1 versus PC2, PC1 versus PC3, and a three-dimensional projection of PC1, PC2, and PC3 are shown. Shaded circles represent 95% confidence intervals based on correlation matrices of the three replicates of each sample. **(D)** Volcano plots of the resistant strains (RES1, RES2, RES3) and tolerant strains (TOL2, TOL5, TOL6) compared to the ancestral strain. Differentially expressed proteins (DEPs) are defined to be those with *p* values below 0.05, and absolute fold change greater than 1.5, corresponding to the colored dots. The protein IDs of the most down-regulated and up-regulated proteins are shown. **(E)** Summary of single point mutations identified in the resistant and tolerant strains, with the respective gene and amino acid substitution. Details about the mutations can be found in Supplementary Table S1. **(F)** The expression level of the genes that are mutated in the evolved strains. Relative abundance of the proteins phosphatidylglycerol lysyltransferase (MprF), ribose-phosphate pyrophosphokinase (Prs), ATP-dependent helicase/deoxyribonuclease subunit B (AddB), serine/threonine protein kinase (PrkC), and 30S ribosomal protein S18 (RpsR) among the ancestral strain and evolved strains, measured by label-free quantitative proteomics using spectral counting, where the *y*-axis is the normalized spectral abundance factor (NSAF) values (mean ± s.e.m., *n* = 3). Strains that express the mutated proteins were marked with red outlines.

The daptomycin-tolerant and resistant strains used in this study were generated from a recent adaptive laboratory evolution (ALE) experiment that mimics clinical conditions (Sulaiman and Lam, 2021b). The tolerant strains were TOL2, TOL5, and TOL6, which have increased survival upon prolonged daptomycin treatment without a change in the MIC, while the resistant strains were RES1, RES2, and RES3, which have elevated MIC toward daptomycin by 3-to 4-fold. Each of these evolved strains bears single point mutations that govern their phenotypes (Supplementary Table S1). While the tolerant strains have mutations in different genes conferring different levels of tolerance toward daptomycin (TOL2 and TOL5 have a mild-tolerance phenotype with ~5-fold increase in survival % after 3 h of treatment, and TOL6 has a high-tolerance phenotype with an over 100-fold increase in survival % after 3 h of treatment), the three resistant strains (RES1, RES2, and RES3) have a single point mutation in the same gene, *mprF*, but in different locations. Figure 1A shows the time-kill curves of the tolerant and resistant strains.

To reveal the alterations in terms of protein expression owing to the single point mutations in the tolerant/resistant strains, we compared the proteome profile of the evolved strains to that of the ancestral strain (the control) in normal growth conditions, without any treatment. Combining all replicates, 1,646, 1,499, 1,451, 1,544, 1,587, 1,489, and 1,302 distinct proteins were identified for ancestral, RES1, RES2, RES3, TOL2, TOL5, and TOL6 strain, respectively (Figure 1B), covering around 60% of the total ~ 2,600 proteins in the proteome of common *S. aureus* strains. Using the protein expression data, we performed a principal component analysis (PCA) to determine possible features that distinguish the ancestral and the tolerant/resistant strains. We observed that all strains except TOL6 were positioned closely, with the 95% confidence interval (CI) ellipse overlapping each other (Figure 1C). TOL6 was positioned uniquely and was separated from the other six strains, indicating that it has the most distinct proteome profile from the rest. Figure 1D shows the volcano plots of fold changes against *p* values (two-tailed *t*-test), highlighting the proteins with different expression levels between the evolved strains and the ancestral strain. The list of DEPs is available in Supplementary Table S2. From the number of DEPs, we observed that the high-tolerance strain TOL6 had the most different protein expression profile from the ancestral strain (354 DEPs). This was consistent with a previous study that reported that a daptomycin-tolerant strain bearing a mutation upstream *pgsA* gene (with a similar survival level to TOL6, >100-fold survival % after 3 h of daptomycin treatment) had significant variations in the proteome profile compared to the ancestral strain (Sulaiman et al., 2021).

### The expression level of the mutated genes in the tolerant and resistant strains

Using the normalized spectral abundance factor (NSAF) values from our proteomics data, we estimated the relative expression level of the proteins encoded by the mutated genes in the evolved strains (Figures 1E,F; Supplementary Table S1). First, all of the resistant strains (RES1, RES2, and RES3) increased the expression of phosphatidylglycerol lysyltransferase, which is the protein encoded by the *mprF* gene, suggesting that the daptomycin resistance phenotype is associated with MprF gain-of-function, consistent with previous reports (Yang et al., 2009; Ernst et al., 2018). In TOL2, proteins for which the genes were mutated, ribose-phosphate pyrophosphokinase (Prs) and ATP-dependent helicase/deoxyribonuclease subunit B (AddB), had significantly lower expression compared to the ancestral and other strains. Interestingly, the expression level of AddB was also lower in another tolerant strain TOL6, indicating that this protein might play a role in daptomycin tolerance. The expression levels of protein serine/threonine-protein kinase (PrkC), which was mutated in TOL5, were similar in all of the strains including TOL5. Although the mutation did not alter the expression level of protein, it might alter the protein function. Finally, the expression level of 30S ribosomal protein S18 (RpsR) was lower in the tolerant TOL6 strain than in the ancestral strain. Interestingly, the expression level of RpsR was also lower in the other resistant/tolerant strains than in the ancestral strain, implying that the down-regulation of RpsR might be a common trend associated with decreased daptomycin susceptibility. The protein of the other mutated gene in TOL6, ProP, was not detected in all of our samples, perhaps because of its low abundance. While the mutations in RES1, RES2, RES3, TOL2, and TOL5 did not alter their growth profile, TOL6 had a significantly higher doubling time than the ancestral strain (Supplementary Figure S1).

### The high-tolerance TOL6 strain has the most alterations in biological processes among the tolerant/resistant strains

**Figure 2:**
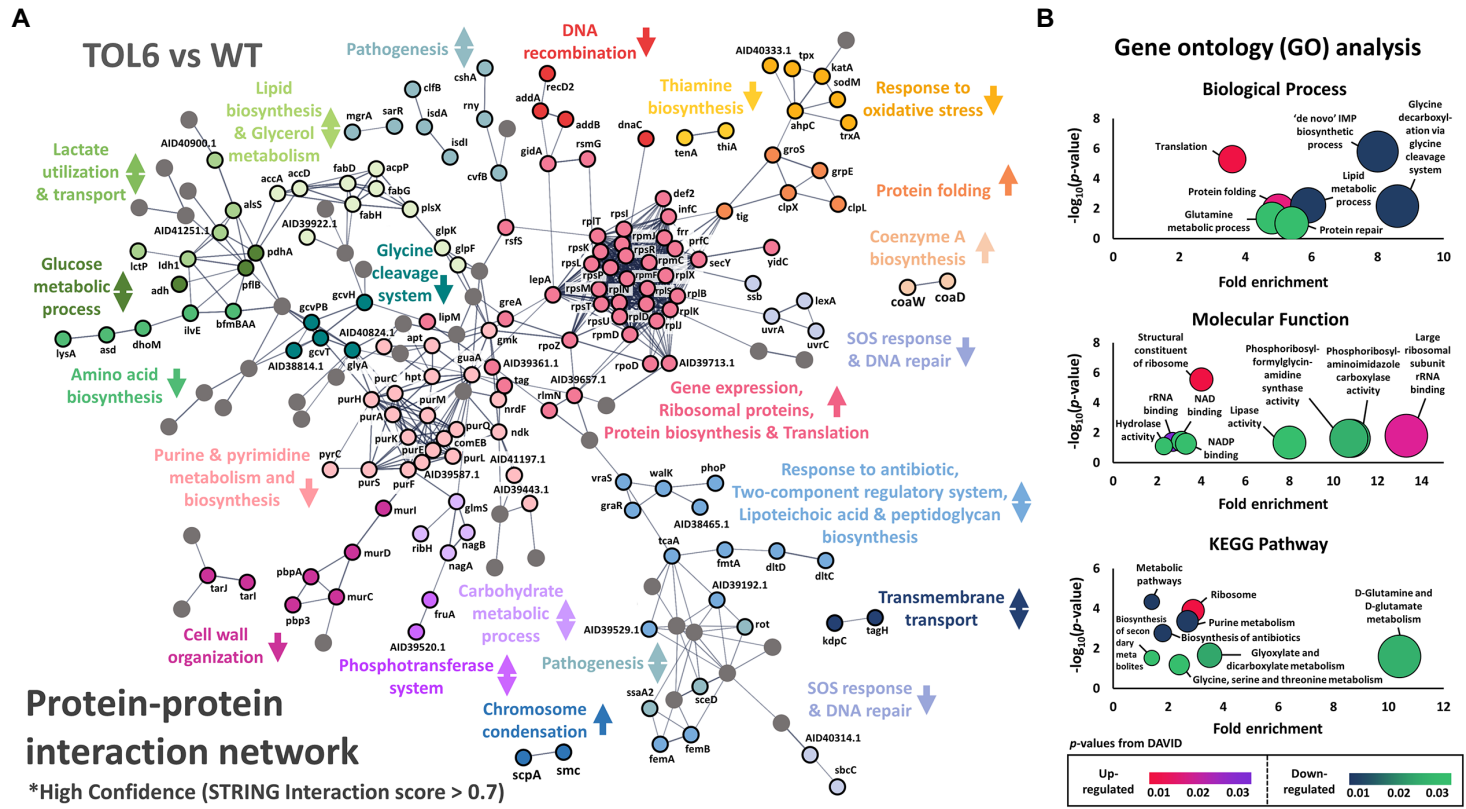
Differentially expressed proteins in the TOL6 strain compared to the ancestral strain. **(A)** Protein–protein interaction network of the DEPs of TOL6 strain compared to the ancestral strain, as predicted by STRING v11.0. The lines represent protein interaction (thicker lines mean higher confidence), and the dots in different colors represent different protein functions. Only high confidence protein–protein interactions are shown (STRING interaction score above 0.7). The arrows beside the protein function indicate the direction of expression of the process (the arrows pointing upwards mean that the process is expressed higher in TOL6, the arrows pointing downwards mean the process is expressed higher in ancestral strain, while arrows pointing both upwards and downwards mean that in a specific process, some proteins are expressed higher in TOL6 and some that are expressed higher in the ancestral strain). Nodes without function enrichment are colored gray. **(B)** Gene Ontology (GO) analysis, classified by the biological process (top) and molecular function (middle), and pathway enrichment study (KEGG; bottom) by DAVID of the DEPs of TOL6 compared to the ancestral strain. Fold enrichment is defined as the ratio of the proportion of the input information to the background information.

The PCA and volcano plots showed that the high-tolerance TOL6 strain had the most different proteome and the highest number of DEPs among the tolerant/resistant strains. Therefore, we were interested in the affected processes due to the mutations it possessed. The protein–protein interaction network of the DEPs in TOL6 is visualized in Figure 2A. There was a wide array of processes that were expressed higher in TOL6 than in the ancestral strain including protein folding, coenzyme A biosynthesis, ribosomal proteins, and chromosome condensation. Those that were expressed lower included DNA recombination, thiamine biosynthesis, response to oxidative stress, SOS response and DNA repair, amino acid biosynthesis, glycine cleavage system, purine and pyrimidine metabolism, and also cell wall organization. The down-regulation of some anabolic processes might be due to the slower growth of the mutant strain (Supplementary Figure S1). For certain processes, such as pathogenesis, response to antibiotic and the two-component system, lipoteichoic acid and peptidoglycan biosynthesis, lipid and glucose metabolism, transmembrane transport, and phosphotransferase system, there was an equal number of proteins that were expressed higher in TOL6 than in the ancestral strain.

Similarly, from the gene ontology (GO) analysis and pathway enrichment study (KEGG) on the DEPs (Figure 2B), we observed that some of the most notable up-regulated processes were protein folding and the expression of ribosomal proteins, while the down-regulated ones were glycine cleavage system, *de novo* inosine monophosphate (IMP) metabolic process, protein repair, lipid metabolism, D-glutamine and D-glutamate metabolism, and glyoxylate and dicarboxylate metabolism. This shutdown of major metabolic processes and the increased expression of ribosomal proteins on TOL6 were not observed in the other tolerant/resistant strains. Instead, it was previously observed in the proteome of *E. coli* (Sulaiman et al., 2018) and *S. aureus* (Huemer et al., 2021) persisters, which are slow-growing cells naturally present in bacterial populations in small quantities and could evade lethal antibiotic treatments. Indeed, *S. aureus* knockouts in glutamate dehydrogenase and other tricarboxylic acid (TCA) cycle enzymes (e.g., 2-oxoketoglutarate dehydrogenase, succinyl coenzyme A synthetase, and fumarase) were previously shown to cause an increased proportion of persister cells and tolerance to different antibiotics (Zalis et al., 2019). More generally, metabolic changes were linked to the formation of persisters by modulating the intracellular level of ATP in *S. aureus* (Conlon et al., 2016). Since TOL6 also had a slow-growing phenotype just like the persisters (Supplementary Figure S1), their similar proteome profile indicated that they might employ a similar approach in surviving antibiotic treatment. Moreover, the higher expression of proteins involved in protein folding in TOL6 was also observed in filamentous *E. coli* persisters from ampicillin treatment (Sulaiman and Lam, 2020b). Stresses such as antibiotic treatment were known to induce protein aggregation. Thus, it was recently postulated that the antibiotic-tolerant persister state is tightly linked to or even driven by protein aggregation (Bollen et al., 2021; Dewachter et al., 2021).

### Affected processes and pathways in the tolerant and resistant strains upon antibiotic treatment

**Figure 3:**
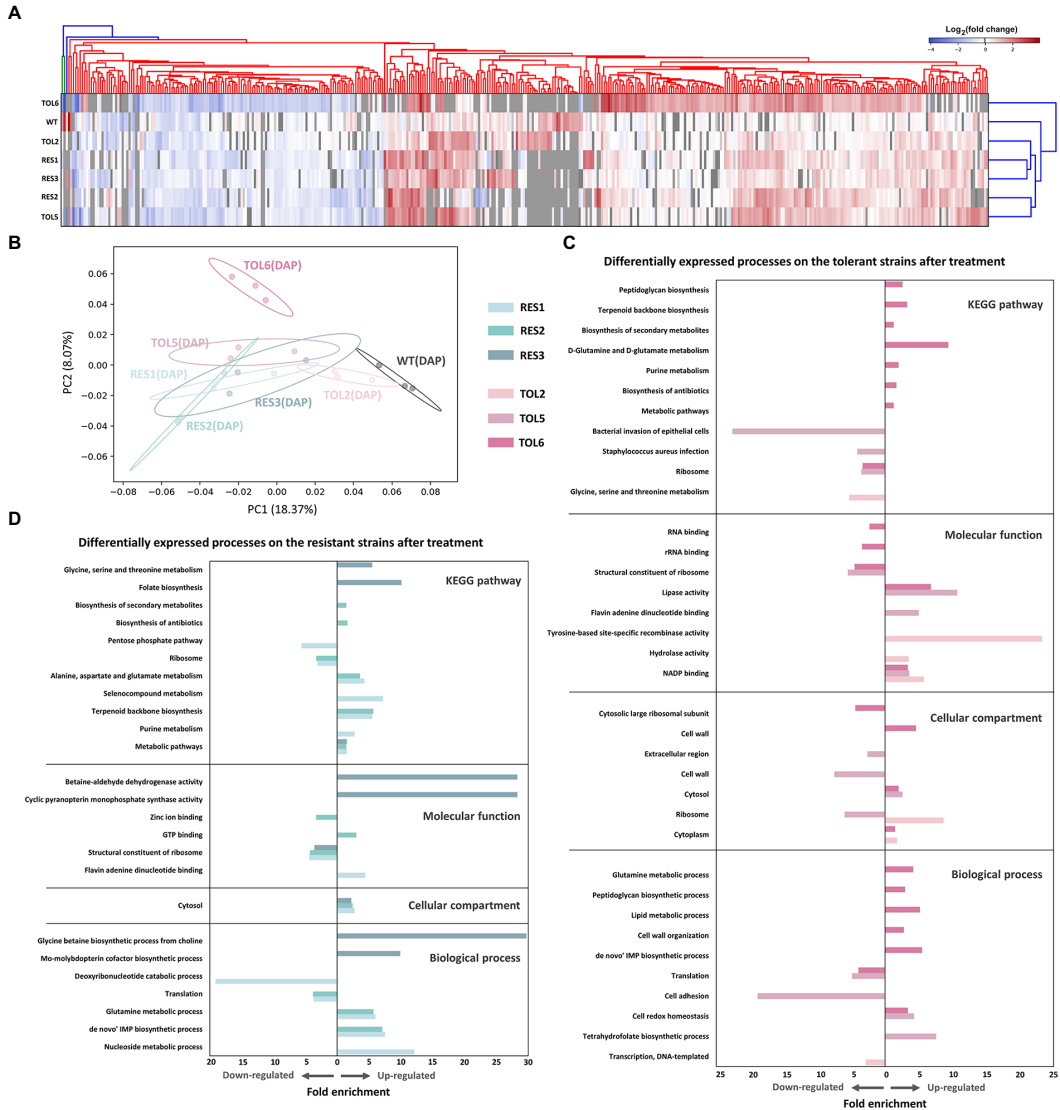
Proteomic response of the tolerant/resistant strains and the ancestral strain upon daptomycin treatment. **(A)** Heatmap of the DEPs across the ancestral strain, resistant strains (RES1, RES2, RES3), and tolerant strains (TOL2, TOL5, TOL6) upon daptomycin treatment compared to the untreated populations. The heatmap is clustered using average linkage hierarchical clustering based on Euclidean distances. The y-axis indicates different strains, and the x-axis represents the DEPs identified across all strains. DEPs that were undetected in specific samples are marked with gray color. **(B)** Principal Component Analysis (PCA) of proteomes of the tolerant/resistant strains and the ancestral strain after daptomycin treatment. Shaded circles represent 95% confidence intervals based on correlation matrices of the three replicates of each sample. **(C,D)** Gene Ontology (GO) analysis and pathway enrichment study (KEGG) by DAVID of the DEPs of the tolerant strains **(C)** and the resistant strains **(D)** after daptomycin treatment compared to those before treatment. Fold enrichment is defined as the ratio of the proportion of the input information to the background information.

Next, we treated the tolerant/resistant strains with a sub-inhibitory concentration of daptomycin to see the effect of antibiotic treatment on the proteomes of the mutant strains. The numbers of protein identified in the treatment groups were similar to the untreated ones (~1,600–1,700 proteins; Supplementary Figure S2a). The number of DEPs in the ancestral strain was the lowest with 79 DEPs, followed by the other resistant/tolerant strains (123, 239, 165, 186, and 122 DEPs for TOL2, TOL5, RES1, RES2, and RES3, respectively), and the highest one was TOL6 with 370 DEPs (Supplementary Figure S2b). Interestingly, the high-tolerance TOL6 strain not only had the most different base-line proteome profile compared to the ancestral strain, but it also had the most significant changes in terms of antibiotic response toward daptomycin. The list of DEPs is available in Supplementary Table S3. From the heat map of the fold changes of all DEPs of the ancestral and evolved strains (Figure 3A), we observed that the DEPs in the TOL2, TOL5, and the resistant strains (RES1, RES2, and RES3) were clustered together, while the DEPs in the ancestral strain and the high-tolerance TOL6 strain were different from the other groups. These suggested that (i) the tolerant/resistant strains have a different antibiotic response compared to the ancestral strain upon daptomycin exposure, but they do share some similarities, and (ii) the high-tolerance TOL6 strain had a different antibiotic response from the ancestral strain and the rest of the tolerant/resistant strains. The latter might also be due to the fact that the proteome of TOL6 was already very different from the rest of the strains even without the addition of antibiotics (Figures 1C,D, 2), and therefore it should adapt differently to antibiotic treatment. Similarly, principal component analysis (PCA) showed that the proteome of the antibiotic-treated samples was positioned similarly, except for TOL6 which was separated from the rest of the groups, especially along PC2 (Figure 3B).

The affected processes and pathways upon antibiotic treatment are shown in Figures 3C,D for the tolerant and resistant strains, respectively. For the tolerant strains, we could see that TOL6 had significant changes. Up-regulated processes include cell redox homeostasis, cell wall organization and peptidoglycan biosynthesis, lipid metabolism, D-glutamine and D-glutamate metabolism, and *de novo* IMP metabolism. Interestingly, all of these processes were expressed lower in TOL6 than in the ancestral strain in the absence of antibiotic (Figure 2). Moreover, we observed that upon antibiotic treatment, TOL5 down-regulated several processes, such as cell adhesion, *S. aureus* infection, and bacterial invasion of epithelial cells, which were related to bacterial virulence and pathogenesis. Ribosomal proteins and protein translation were down-regulated not only in the TOL5 and TOL6 upon antibiotic treatment (Figure 3C), but also in the resistant strains (Figure 3D). This might be related to the fact that daptomycin posed a certain degree of inhibition against protein synthesis (Heidary et al., 2018). In the resistant strain RES3, one of the most apparent up-regulated processes was the glycine betaine biosynthesis process. An increased level of glycine betaine has been shown to be associated with daptomycin resistance (Song et al., 2013). A study examining the transcriptome of a daptomycin-resistant MRSA strain revealed an accumulation of glycine betaine within the cells, coupled with the up-regulation of choline transporter (*cudT*), choline dehydrogenase (*betA*), glycine betaine aldehyde dehydrogenase (*gbsA*), *opuD2*, and *proP* genes (Song et al., 2013). From our proteomics data, choline dehydrogenase was up-regulated by 2.0-, 2.5-, and 1.7-folds in RES1, RES2, and RES3, respectively, betaine aldehyde dehydrogenase was up-regulated by 2.1-and 2.0-folds in RES2 and RES3, respectively, and probable glycine dehydrogenase subunit 1 was up-regulated by 1.5-folds in both RES1 and RES3. Moreover, cell wall and membrane-active antibiotics such as daptomycin was known to cause oxidative stress and protein aggregation and misfolding, as revealed by the induction of molecular chaperones (Utaida et al., 2003; Wilkinson et al., 2005; Kohanski et al., 2008; Sulaiman and Lam, 2021b). Glycine betaine was reported to promote normal protein folding in stressed cells, and its accumulation helped bacteria to survive antibiotic assault (Arakawa and Timasheff, 1991; Record et al., 1998). It is worth noting that the *proP* gene that expresses proline/betaine transporter was also mutated in TOL6, leading to a truncation of 22 amino acids in the corresponding protein and a reduced sensitivity toward daptomycin (Supplementary Table S1).

### Cross-comparison of multiple mutants highlighted key proteins that might be important for their phenotypes

**Figure 4:**
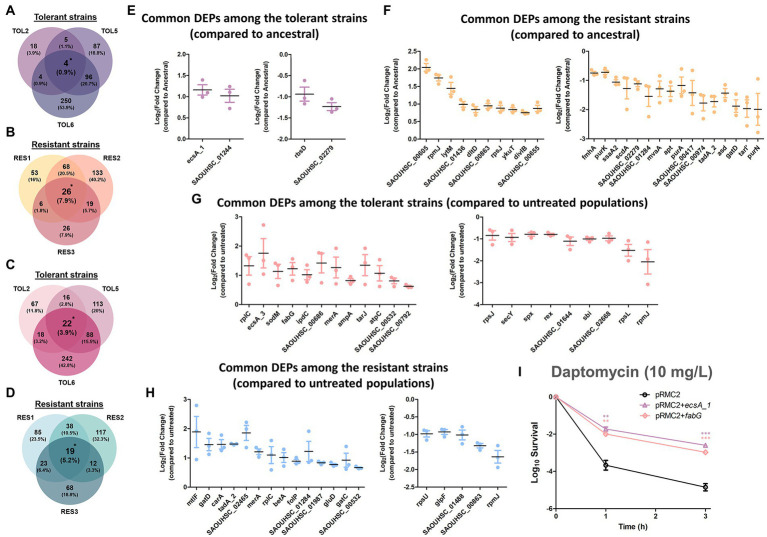
Commonly expressed DEPs among the tolerant and resistant strains. **(A–D)** Venn diagrams of the DEPs in the three tolerant strains compared to the ancestral strain **(A)**, the three resistant strains compared to the ancestral strain **(B)**, the three tolerant strains compared to the untreated populations **(C)**, the three resistant strains compared to the untreated populations **(D)**. DEPs shared between all three strains were marked with asterisks. **(E-H)** Fold changes in the overlapped DEPs among the three tolerant strains compared to the ancestral strain **(E)**, the three resistant strains compared to the ancestral strain **(F)**, the three tolerant strains compared to the untreated populations **(G)**, the three resistant strains compared to the untreated populations (**H**; mean ± s.e.m., *n* = 3). The left figures show up-regulated proteins, and the right figures show down-regulated proteins. **(I)** Gene overexpression of the commonly expressed DEPs among the tolerant strains. Mutants of MRSA strain harboring empty pRMC2 plasmid, pRMC2 + *ecsA1* plasmid, and pRMC2 + *fabG* plasmid were constructed and subjected to tolerance assay. Survival of the overexpressed mutants under daptomycin treatment (10 mg/l) is shown (mean ± s.e.m., *n* = 3). Significance of difference from the wild-type bearing empty pRMC2 plasmid: ns, not significant, **P < 0.01, ***P < 0.001 (two-tailed *t*-test with unequal variances). For strains bearing pRMC2 plasmids, 0.2 μg/ml anhydrotetracycline was added to induce the expression of overexpressed genes.

Besides looking at the DEPs in individual strains, we sought to determine if there were any common DEPs across the tolerant/resistant strains that may act as the key players of the tolerance/resistance phenotype. This cross-comparison strategy of the proteome profile has been previously employed in *E. coli* tolerant strains and was proven to be effective in highlighting the key proteins for tolerance (Sulaiman and Lam, 2020a). By comparing each of the evolved tolerant/resistant strains to the ancestral strain, we identified 4 and 26 DEPs that were shared among the three tolerant strains (TOL2, TOL5, TOL6) and among the three resistant strains (RES1, RES2, RES3), respectively (Figures 4A,B). The common DEPs with the corresponding expression level (in terms of fold changes) are shown in Figures 4E,F for the tolerant and resistant strains, respectively. In the resistant strains, we found that most of the common DEPs were cell division and cell wall-related proteins. The up-regulated proteins were: autolysin glycyl-glycine endopeptidase LytM (known to cleave the polyglycine interpeptide bridges of the cell wall peptidoglycan), protein DltD (involved in the D-alanylation of lipoteichoic acid which influences the net charge of the cell wall), cell division protein DivIB (involved in stabilizing or promoting the assembly of the division complex). The down-regulated proteins were: cell wall-related protein ScdA (involved in the repair of iron–sulfur clusters damaged by oxidative and nitrosative stress conditions), ribitol-5-phosphate cytidylyltransferase 2 (TarI), aminoacyltransferase FemA (FmhA), lipid II isoglutaminyl synthase subunit GatD, staphylococcal secretory antigen SsaA2, adenine phosphoribosyltransferase (Apt, involved in purine metabolism), and proteins PurA, PurK, and PurN which are all part of the purine biosynthetic pathway. For the common DEPs among the tolerant strains, all of them had a higher expression of the ABC transporter domain-containing protein (EcsA1) and a lower expression of protein RbsD, which catalyzes the interconversion of beta-pyran and beta-furan forms of D-ribose.

By comparing each of the antibiotic-treated evolved tolerant/resistant strains to the untreated cultures of the same strain, we identified 22 and 19 DEPs that were shared among the three tolerant strains and among the three resistant strains, respectively (Figures 4C,D). Figures 4G,H shows the common DEPs with the corresponding expression level (in terms of fold changes) for the tolerant and resistant strains, respectively. Several proteins that we previously observed to have a lower expression in the resistant strains (Figure 4F, right) became up-regulated after antibiotic treatment, such as lipid II isoglutaminyl synthase subunit GatD, CMP/dCMP-type deaminase domain-containing protein (TadA2), and putative aluminum resistance protein (SAOUHSC_01284). In addition, we also observed that mannitol-specific phosphotransferase enzyme IIA component (MtlF, part of the phosphotransferase system), and oxygen-dependent choline dehydrogenase (BetA, involved in the biosynthesis of the osmoprotectant glycine betaine) were commonly up-regulated among the resistant strains. This reinforced the notion that glycine betaine is important for resistance against daptomycin stress as previously discussed. In the tolerant strains, we observed that another ABC transporter domain-containing protein (EcsA3) was commonly up-regulated, similar to what we observed from the tolerant strains compared to the ancestral strain in the absence of antibiotic (Figure 4E), suggesting that transporters might play a role in daptomycin tolerance. Other up-regulated proteins include superoxide dismutase [Mn/Fe] 2 (SodM) that destroys superoxide anion radicals and maintains cell viability during the late-exponential and stationary phase, 3-oxoacyl-[acyl-carrier-protein] reductase (FabG) that catalyzes the first reductive step in the elongation cycle of fatty acid biosynthesis, ribulose-5-phosphate reductase 1 (TarJ) which takes part in cell wall biogenesis, and ATP synthase epsilon chain (AtpC) that produces ATP from ADP in the presence of a proton gradient across the membrane.

For the commonly down-regulated proteins among the tolerant strains, they were protein translocase subunit SecY (involved in protein transport), global transcriptional regulator Spx (a master regulator involved in stress response), redox-sensing transcriptional regulator Rex (known to modulate transcription in response to changes in cellular NADH/NAD^+^ redox state), lactamase B domain-containing protein (SAOUHSC_01644), and immunoglobulin-binding protein Sbi. Interestingly, the last two proteins were also down-regulated in two other daptomycin-tolerant strains in our previous study (one with a high-tolerance level like TOL6 and one with a mild-tolerance level like TOL2 and TOL5) in the absence and presence of daptomycin (Sulaiman et al., 2021). Combining our two studies, we were struck by the finding that these two proteins had the same trend of lower expression in five different daptomycin-tolerant strains bearing completely different point mutations, and therefore might serve as tolerance markers of MRSA. Lactamase B domain-containing protein has a homologous sequence to β-lactamases, enzymes conferring resistance to β-lactams, whereas Sbi is anchored to the cell envelope by binding to the lipoteichoic acid (LTA). Since an LTA-defective mutant of *S. aureus* reduces Sbi levels (Smith et al., 2012), it is possible that daptomycin-tolerant strains, in general, have a reduced number of LTA molecules anchored in the cell wall.

### Impact of *ecsA1* and *fabG* overexpression on the daptomycin tolerance phenotype

From the list of commonly expressed DEPs that serve as potential key players of tolerance (Figure 4), we selected EcsA1 and FabG for a follow-up study in gene overexpression analysis using the expression vector pRMC2. The fold change of EcsA1 in the tolerant strains compared to the ancestral strain are 1.99, 2.13, and 2.63 for TOL2, TOL5, and TOL6, respectively (Supplementary Table S2), and the fold change of FabG in the tolerant strains upon daptomycin treatment compared to the untreated ones are 1.77, 2.95, and 2.43 for TOL2, TOL5, and TOL6, respectively (Supplementary Table S3). Verification of the gene overexpression was performed using RT-qPCR, as shown in Supplementary Figure S3.

EcsA1 is an ABC transporter domain-containing protein that was up-regulated in all of our untreated tolerant strains, whereas FabG (3-oxoacyl-[acyl-carrier-protein] reductase) was up-regulated in all of the tolerant strains upon treatment with daptomycin, suggesting that this protein might be important for the adaptation of the tolerant cells toward daptomycin. Indeed, overexpression of EcsA1 and FabG led to increased daptomycin tolerance in MRSA with ~150-and ~ 60-fold increase in survival after 3 h of treatment compared to the wild-type bearing empty pRMC2 plasmid (Figure 4I), without any increase in the MIC (Supplementary Table S4). Although the ABC transporter system plays a role in transporting toxic compounds such as toxins, drugs, and detergents (Li et al., 2016), the function of EcsA1 in *S. aureus* antibiotic tolerance remained largely uncharacterized and requires further investigation. Certainly, the significant increase in survival to daptomycin upon the overexpression of this gene testifies to its importance for the cells’ tolerance phenotype. On the other hand, up-regulation of FabG, a key enzyme in fatty acid biosynthesis, is expected to alter lipid metabolism in the cells, which was also previously linked to decreased susceptibility toward daptomycin (Hofer, 2016; Hines et al., 2017; Lee et al., 2019). For instance, *S. aureus* inactivates daptomycin by releasing membrane phospholipids into the extracellular space, thereby sequestering daptomycin and preventing it from inserting into the bacterial membrane (Pader et al., 2016). Strains with different genetic backgrounds may exhibit different contributions of phospholipid shedding and hence have different tolerance levels to daptomycin (Shen et al., 2021). Indeed, in our previous study, we found that reduced daptomycin tolerance in MRSA was associated with a reduced lipid metabolic process (Sulaiman et al., 2021). Besides, through integrated multi-omics, virtual screening, and molecular docking analysis, Rahman et al. suggested several potential drug targets against *S. aureus,* including several *fab* genes which are responsible for fatty acid synthesis, such as malonyl CoA-acyl carrier protein transacylase FabD, 3-oxoacyl-[acyl-carrier-protein] synthase 3 FabH, and enoyl-[acyl-carrier-protein] reductase [NADPH] FabI.

Overall, we showed that overexpression of *ecsA1* and *fabG* increased daptomycin tolerance by 150-and 60-fold, respectively, suggesting that ABC transporter system and fatty acid metabolism play key roles in modulating daptomycin tolerance. These experiments also demonstrated the utility of our strategy of cross comparing the proteomes of distinct resistant/tolerant mutants in identifying novel gene and protein candidates relevant to these phenotypes.

### The daptomycin-resistant and tolerant MRSA strains modulate their cell wall differently

**Figure 5:**
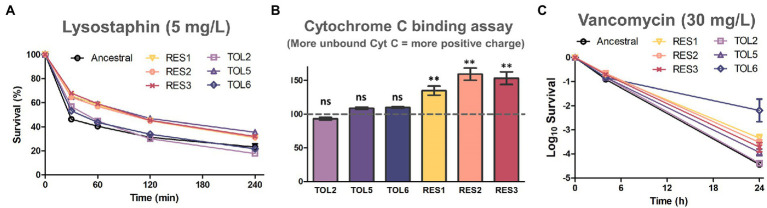
Assays to assess cell wall modifications on the evolved strains. **(A)** Lysostaphin lysis assay in the ancestral strain and evolved strains. Cells were treated with 5 mg/l of lysostaphin, incubated at 37°C, and the decrease in OD_600_ was monitored over time (mean ± s.e.m., *n* = 3). **(B)** Binding of positively charged cytochrome *c* to the ancestral and evolved strains. The y-axis shows the percentage of unbound cytochrome c in comparison with the ancestral strain (marked with the horizontal dashed line; mean ± s.e.m., *n* = 3). Significance of difference from the ancestral strain: ns, not significant, **P< 0.01 (two-tailed *t*-test with unequal variances). **(C)** Time-kill curve of exponential phase ancestral strain and evolved strains with 30 mg/l of vancomycin (mean ± s.e.m., *n* = 3).

Lastly, we wanted to investigate whether the tolerant and resistant strains possessed modifications in their cell wall properties, motivated by our observation that many common DEPs among the resistant strains were related to the cell wall, such as LytM, DltD, GatD, DivIB, FmhA, ScdA, and tarI (Figure 4). In addition, the expression of the MprF protein which was mutated in the resistant strains (coding for an enzyme related to cell wall modifications) was also increased in the resistant strains (Figure 1F). We exposed the resistant strains to lysostaphin, which is an endopeptidase that cleaves the cross-linking pentaglycine bridges on the peptidoglycan layer, and found that all of the resistant strains had higher survival than the ancestral strain (Figure 5A). This indicated that the resistant strains had modifications in the cell wall peptidoglycan. Interestingly, TOL5, which possessed a mutation in the *prkC* gene, also had an increased survival toward lysostaphin. This was consistent with a previous study that shows an *S. aureus* Δ*prkC* mutant has cell wall modifications and increased resistance to Triton-X100 and fosfomycin (Débarbouillé et al., 2009). Besides, several lines of indirect evidence have also suggested that *prkC* contributes to *S. aureus* cell wall synthesis. In *S. aureus, prkC* phosphorylated the response regulator GraR of the two-component system GraRS, and the phosphorylated GraR increased the expression of the *dlt* operon, thus triggering modifications of cell wall teichoic acids (Manuse et al., 2016). The other two tolerant strains, TOL2 and TOL6, had similar survival profiles to the ancestral strain under lysostaphin treatment.

Next, we tested whether alteration of cell surface charge played a role in repelling daptomycin by quantifying the association of the highly cationic cytochrome *c* molecule to the cell’s surface (Figure 5B). We observed that all of the resistant strains had a higher percentage of unbound cytochrome *c* than the ancestral strain, suggesting that their surface charge was more positive. This is likely because the nonsynonymous gain-of-function mutations in *mprF* on the resistant strains increased the production of positively charged lysyl-phosphatidylglycerol (LysPG), enhanced the net positive surface charge, and ultimately reduced daptomycin binding (Yang et al., 2009). On the other hand, the increased tolerance of TOL2, TOL5 and TOL6 was not linked to surface charge alteration, as no significant difference in cytochrome *c* binding was observed for these strains relative to the ancestral strain.

Besides daptomycin, another commonly used antibiotic in clinics to treat MRSA is vancomycin, which inhibits cell wall synthesis by binding to the D-Ala-D-Ala terminal of growing peptide chains. We observed that all of the tolerant strains TOL2, TOL5, and TOL6 had a slight increase in the MIC toward vancomycin, whereas the resistant strains RES1, RES2, and RES3 had elevated MICs toward vancomycin (Supplementary Table S5). Under a prolonged treatment with a lethal concentration of vancomycin, all of the evolved strains except TOL2 had a higher survival after 24 h (Figure 5C), indicating that while the resistant strains had modifications in their peptidoglycan and a more positive cell surface charge, the tolerant strains possessed other cell wall changes that might also reduce the effectiveness of vancomycin, but the peptidoglycan does not seem to be involved. Also, while TOL5, RES1, RES2, and RES3 had a mild increase in survival to vancomycin (3.5-to 14-fold) compared to the ancestral strain, the survival of the high-tolerance strain TOL6 was 467-fold higher. This extreme cross-tolerance observed in TOL6 might also be due to their slower growth (Supplementary Figure S1), reminiscent of the characteristic of persister cells that evade antibiotics by inactivating their targets (Lewis, 2007). In this case, TOL6 had a much lower expression of proteins involved in cell wall synthesis than the ancestral and other strains (Figure 2; Supplementary Table S2), which explains why it had a much higher survival toward vancomycin.

Daptomycin disrupts multiple aspects of the cell membrane and inhibits DNA, RNA, and protein synthesis, eventually leading to cell death (Gray and Wenzel, 2020). Although there was evidence that daptomycin did not directly inhibit cell wall synthesis, other studies continued to find cell wall-related phenotypes and induction of cell wall stress stimulons upon daptomycin treatment. Studies have reported that daptomycin acted synergistically with beta-lactam antibiotics (Rand and Houck, 2004; Renzoni et al., 2017; Ye et al., 2019), and proteomics studies similar to ours have indicated that daptomycin induces cell wall stress response proteins in *S. aureus* (Ma et al., 2017) and other organisms such as *B. subtilis* (Wecke et al., 2009; Wenzel et al., 2012; Müller et al., 2016). Consistent with previous studies, we also found that the tolerant and resistant strains possessed alterations in their cell wall properties (Figure 5; Supplementary Table S5), although it remains unclear how exactly these cell wall phenotypes led to decreased daptomycin susceptibility.

## Conclusion

In conclusion, we performed a deep proteome profiling of different daptomycin-tolerant and resistant MRSA strains and compared their protein expression profiles. Overall, we revealed proteome alterations associated with the specific tolerance/resistant mutations, showed how the different strains responded to antibiotic treatment and highlighted the unique processes associated with each of the phenotypes, and pointed out key proteins for daptomycin tolerance and resistance in MRSA. Through different cell wall assays, we showed that the tolerant and resistant strains modulated their cell wall differently. While the resistant strains have modifications in their cell wall peptidoglycan and have a more positive surface charge to repel daptomycin binding, tolerant strains possessed other cell wall modifications that do not involve peptidoglycan or surface charge alterations. We believe that our work is a clear step forward into understanding the different daptomycin tolerance and resistance phenotypes in MRSA, and the data generated from our proteomics study would be useful for other researchers in the field.

## Data availability statement

The mass spectrometry proteomics data have been deposited to ProteomeXchange via the PRIDE repository with the dataset identifier PXD026741. Whole-genome sequence data of the evolved strains is available in the BioProject database (NCBI) under the accession number PRJNA724993.

## Author contributions

JS: conceptualization, methodology, and formal analysis. JS and LL: investigation. JS and HL: writing—original draft. JS, HL, LL, and P-YQ: writing—review and editing. HL: funding acquisition. HL and P-YQ: supervision. All authors contributed to the article and approved the submitted version.

## Funding

The authors acknowledge the support from Hong Kong Branch of Southern Marine Science and Engineering Guangdong Laboratory (Guangzhou; SMSEGL20SC01), Ministry of Science and Technology (MOST19SC06), and Research Grant Council (Grant Nos. 16102821, 16307620, R5013-19, and C6026-19G-A), of the Hong Kong Special Administrative Region, China.

## Conflict of interest

The authors declare that the research was conducted in the absence of any commercial or financial relationships that could be construed as a potential conflict of interest.

## Publisher’s note

All claims expressed in this article are solely those of the authors and do not necessarily represent those of their affiliated organizations, or those of the publisher, the editors and the reviewers. Any product that may be evaluated in this article, or claim that may be made by its manufacturer, is not guaranteed or endorsed by the publisher.

## Footnotes

^1^ http://n2t.net/addgene:68940

## Supplementary material

SUPPLEMENTARY FIGURE S1 Doubling times of the ancestral strain and TOL6. The values were extracted from the fit to the exponential growth phase (mean ± s.e.m., *n* = 3). Significance of difference with the ancestral: ****P* < 0.001, (two-tailed Student’s *t*-test).

SUPPLEMENTARY FIGURE S2 Proteome comparison of the strains before and after daptomycin treatment. **(a)** Venn diagrams for proteome comparison of the ancestral strain, resistant strains (RES1, RES2, RES3), and tolerant strains (TOL2, TOL5, TOL6) upon daptomycin treatment with those before treatment. **(b)** Volcano plots of the ancestral strain, resistant strains (RES1, RES2, RES3), and tolerant strains (TOL2, TOL5, TOL6) upon daptomycin treatment compared to those before treatment. Differentially expressed proteins (DEPs) are defined to be those with *p*-values below 0.05, and absolute fold change greater than 1.5, corresponding to the colored dots. The protein IDs of the most down-regulated and up-regulated proteins are shown.

SUPPLEMENTARY FIGURE S3 Quantitative real-time PCR validation of the gene overexpression using the expression vector pRMC2. Relative expression levels of *fabG* and *ecsA1* genes on the overexpressed mutants and strain bearing empty pRMC2 plasmid were normalized to that of the reference gene *gyrA* (mean ± s.e.m., *n* = 4).

SUPPLEMENTARY TABLE S1 List of single point mutations on the evolved strains.

SUPPLEMENTARY TABLE S2 List of the differentially expressed proteins in the tolerant (TOL2, TOL5, TOL6) and resistant strains (RES1, RES2, RES3) compared to the ancestral strain.

SUPPLEMENTARY TABLE S3 List of the differentially expressed proteins in the ancestral strain, tolerant (TOL2, TOL5, TOL6), and resistant strains (RES1, RES2, RES3) upon daptomycin treatment compared to the untreated ones.

SUPPLEMENTARY TABLE S4 MIC values of mutants of MRSA strain harboring empty pRMC2 plasmid, pRMC2+*ecsA1* plasmid, and pRMC2+*fabG* plasmid.

SUPPLEMENTARY TABLE S5 MIC values of the ancestral strain and evolved strains to daptomycin and vancomycin.

SUPPLEMENTARY TABLE S6 Bacterial strains, plasmids, and primers used in this study.
