# Supplementary figures and images for: Proteome profiling of evolved methicillin-resistant Staphylococcus aureus strains with distinct daptomycin tolerance and resistance phenotypes
Source: Front Microbiol. 2022 Aug 4;13:970146. doi: 10.3389/fmicb.2022.970146 (PMC9386379; doi:10.3389/fmicb.2022.970146)

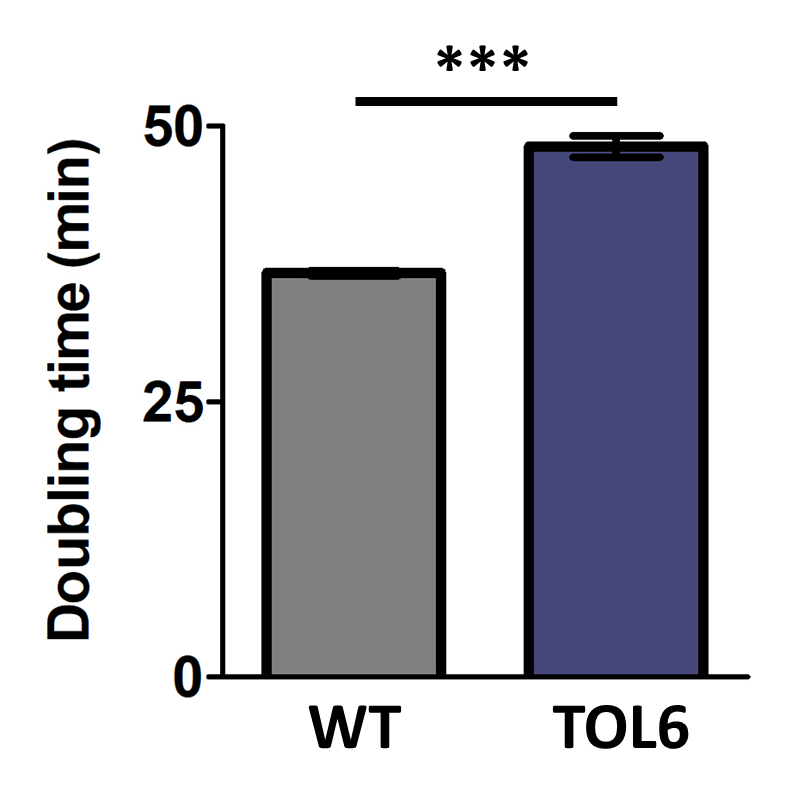

Supplement: SUPPLEMENTARY FIGURE S1 — Doubling times of the ancestral strain and TOL6. The values were extracted from the fit to the exponential growth phase (mean ± s.e.m., n = 3). Significance of difference with the ancestral: ***P < 0.001, (two-tailed Student’s t-test). [file Image_1.TIF]

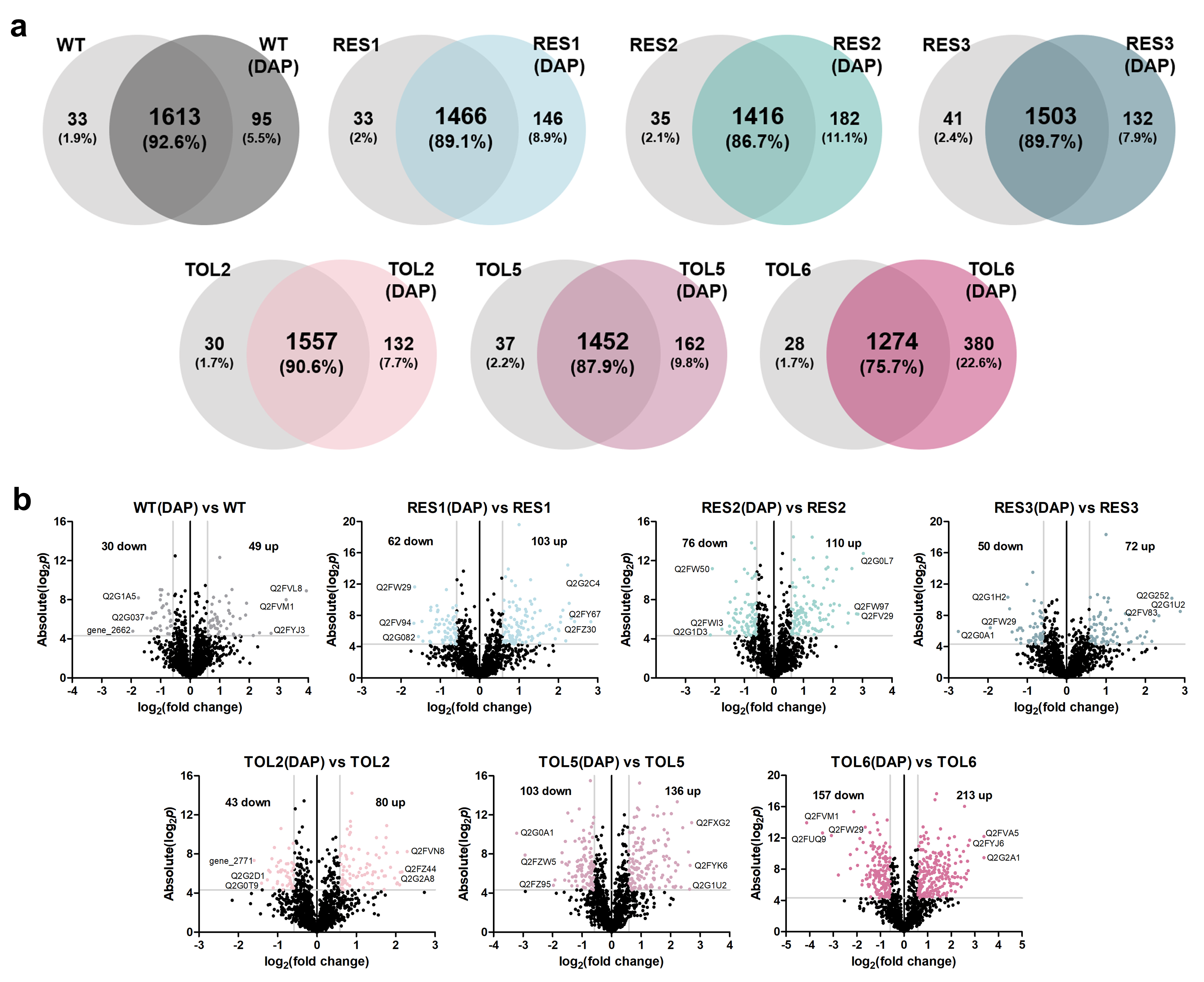

Supplement: SUPPLEMENTARY FIGURE S2 — Proteome comparison of the strains before and after daptomycin treatment. (a) Venn diagrams for proteome comparison of the ancestral strain, resistant strains (RES1, RES2, RES3), and tolerant strains (TOL2, TOL5, TOL6) upon daptomycin treatment with those before treatment. (b) Volcano plots of the ancestral strain, resistant strains (RES1, RES2, RES3), and tolerant strains (TOL2, TOL5, TOL6) upon daptomycin treatment compared to those before treatment. Differentially expressed proteins (DEPs) are defined to be those with p-values below 0.05, and absolute fold change greater than 1.5, corresponding to the colored dots. The protein IDs of the most down-regulated and up-regulated proteins are shown. [file Image_2.TIF]

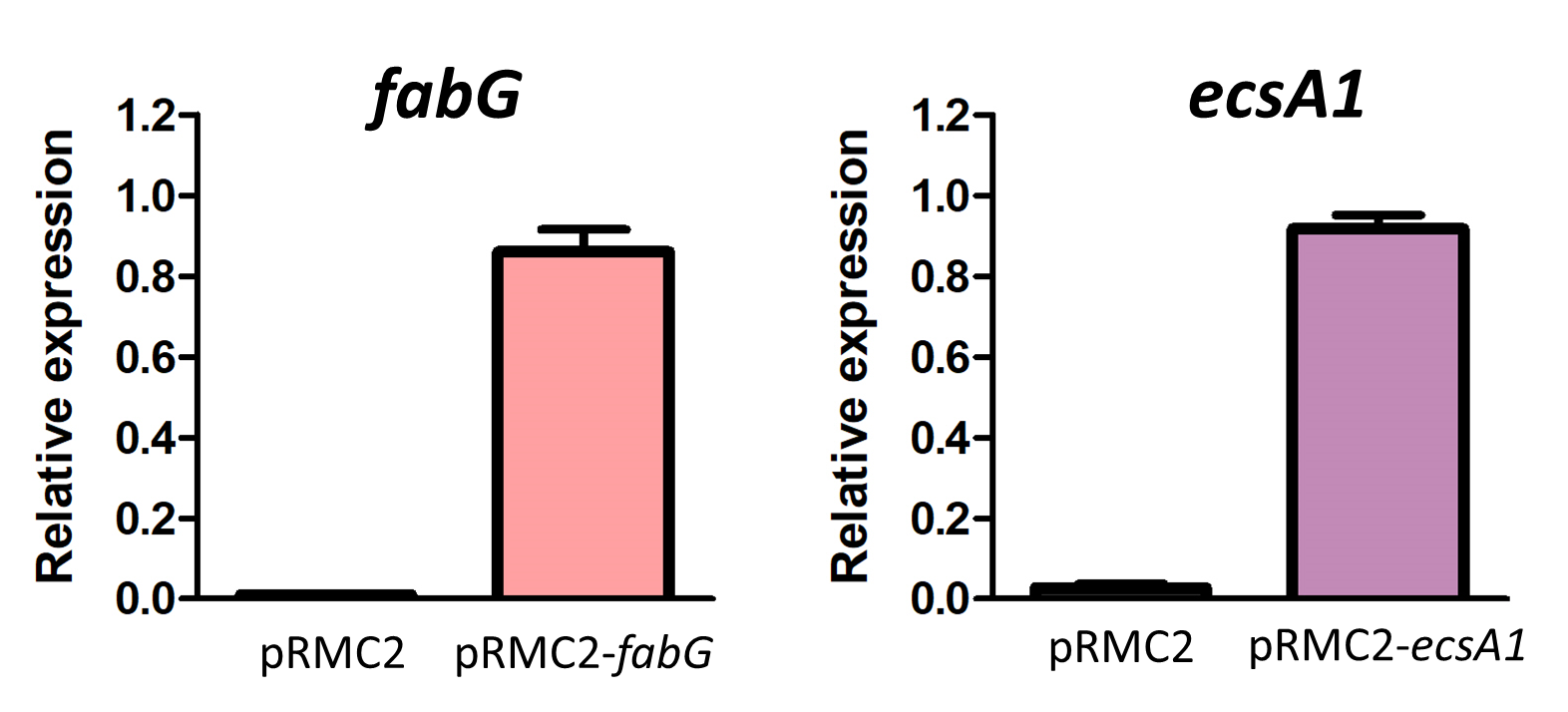

Supplement: SUPPLEMENTARY FIGURE S3 — Quantitative real-time PCR validation of the gene overexpression using the expression vector pRMC2. Relative expression levels of fabG and ecsA1 genes on the overexpressed mutants and strain bearing empty pRMC2 plasmid were normalized to that of the reference gene gyrA (mean ± s.e.m., n = 4). [file Image_3.TIF]
