# Supplementary material for: Proteome profiling of evolved methicillin-resistant Staphylococcus aureus strains with distinct daptomycin tolerance and resistance phenotypes
Source: Front Microbiol. 2022 Aug 4;13:970146. doi: 10.3389/fmicb.2022.970146 (PMC9386379; doi:10.3389/fmicb.2022.970146)
Supplement: SUPPLEMENTARY TABLE S1 — List of single point mutations on the evolved strains. [file Table_1.DOCX]

| **Strain** | **Genomic position** | **Mutation** | **Amino acid substitution** | **Gene** | **Annotation** |
| --- | --- | --- | --- | --- | --- |
| **TOL2** | 1,138,515 | G > A | Ser791Leu | *addB* | ATP-dependent helicase/deoxyribonuclease subunit B |
|  | 1,568,459 | G > A | Ser262Leu | *prs* | Ribose-phosphate pyrophosphokinase |
| **TOL5** | 859,633 | C > A | Gly18Val | *prkC* | Serine/threonine-protein kinase |
| **TOL6** | 1,464,075 | G > A | Gln445**Stop** | *proP* | Proline/betaine transporter |
|  | 1,683,025 | A > G | **Stop**81Gln | *rpsR* | 30S ribosomal protein S18 |
| **RES1** | 691,770 | C > A | Arg50Leu | *mprF* | Phosphatidylglycerol lysyltransferase |
| **RES2** | 690,909 | G > A | Ser337Leu |  |  |
| **RES3** | 690,644 | T > G | Leu425Phe |  |  |

* The raw data of the whole-genome sequencing could be found in the BioProject database (NCBI) under the accession number PRJNA724993.
