# Supplementary material for: Proteome profiling of evolved methicillin-resistant Staphylococcus aureus strains with distinct daptomycin tolerance and resistance phenotypes
Source: Front Microbiol. 2022 Aug 4;13:970146. doi: 10.3389/fmicb.2022.970146 (PMC9386379; doi:10.3389/fmicb.2022.970146)
Supplement: SUPPLEMENTARY TABLE S4 — MIC values of mutants of MRSA strain harboring empty pRMC2 plasmid, pRMC2+ecsA1 plasmid, and pRMC2+fabG plasmid. [file Table_4.DOCX]

| **Strain** | **MIC (mg/L)** |
| --- | --- |
| WT | 1 |
| pRMC2 | 1 |
| pRMC2+*ecsA_1* | 1 |
| pRMC2+*fabG* | 1 |
