# Supplementary material for: Proteome profiling of evolved methicillin-resistant Staphylococcus aureus strains with distinct daptomycin tolerance and resistance phenotypes
Source: Front Microbiol. 2022 Aug 4;13:970146. doi: 10.3389/fmicb.2022.970146 (PMC9386379; doi:10.3389/fmicb.2022.970146)
Supplement: SUPPLEMENTARY TABLE S5 — MIC values of the ancestral strain and evolved strains to daptomycin and vancomycin. [file Table_5.DOCX]

| **Strain** | **MIC (mg/L)** | |
| --- | --- | --- |
|  | **DAP** | **VAN** |
| Ancestral WT | 1 | 1 |
| TOL2 | 1 | 2 |
| TOL5 | 1 | 2 |
| TOL6 | 1 | 2 |
| RES1 | 4 | 4 |
| RES2 | 4 | 4 |
| RES3 | 3 | 2 |
