# Supplementary material for: Proteome profiling of evolved methicillin-resistant Staphylococcus aureus strains with distinct daptomycin tolerance and resistance phenotypes
Source: Front Microbiol. 2022 Aug 4;13:970146. doi: 10.3389/fmicb.2022.970146 (PMC9386379; doi:10.3389/fmicb.2022.970146)
Supplement: SUPPLEMENTARY TABLE S6 — Bacterial strains, plasmids, and primers used in this study. [file Table_6.DOCX]

| **Strains** | **Description** | **Reference** |
| --- | --- | --- |
| **MRSA** | | |
| ATCC43300 | Wild-type methicillin-resistant *S. aureus* | ATCC |
| ATCC43300 pRMC2 | ATCC43300 with empty pRMC2 | This study |
| ATCC43300 pRMC2-*ecsA1* | ATCC43300 with pRMC2-*ecsA1* | This study |
| ATCC43300 pRMC2-*fabG* | ATCC43300 with pRMC2-*fabG* | This study |
| **Plasmids** | **Description** | **Reference** |
| pRMC2 | Tetracycline-inducible expression vector | Corrigan & Foster (2009) Plasmid, 61:126-129 |
| pRMC2-*ecsA1* | pRMC2 with *ecsA1* | This study |
| pRMC2-*fabG* | pRMC2 with *fabG* | This study |
| **Primers** | **Sequence** | **Description** |
| pRMC2-F | aatcctgttaccagtggctg | For checking/ confirmation of pRMC2 |
| pRMC2-R | gatacctgtccgcctttctc |  |
| pRMC2-Tet-R | ggcgagtttacgggttgtta |  |
| *ecsA1*-sacI-F | CGAGCTCatggatgttttaacaatagaacat | Amplification of *ecsA1* fragment |
| *ecsA1*-kpnI-R | GGTACCCCttatgcttgatcctcccttt |  |
| *ecsA1*-kpnI-R1 | GGTACCttatgcttgatcctcccttt |  |
| *fabG*-sacI-F | CGAGCTCatgactaagagtgctttagtaaca | Amplification of *fabG* fragment |
| *fabG*-kpnI-R | GGTACCCCttacatgtacattccaccattt |  |
| *fabG*-kpnI-R1 | GGTACCttacatgtacattccaccattt |  |
| *ecsA1*-rt-F | agcaatatcggtgcattgat | RT-qPCR |
| *ecsA1*-rt-R | tccatatgaagttgtgagac |  |
| *fabG*-rt-F | caagcaaactatgttgcaac |  |
| *fabG*-rt-R | agctcatcacttaaagcatc |  |
| *gyrA*-rt-F | gcggttgtaaatgatgaaaca |  |
| *gyrA*-rt-R | catcatcatctgatgattgtt |  |
